# Nursing vigilance in preventing catheter fragment migration: a dual case report of peripheral venous catheter fractures in pediatric practice

**DOI:** 10.3389/fped.2025.1638983

**Published:** 2025-07-16

**Authors:** Lingling Pu, Youcheng Zhang, Weibing Chen, Hongyan Meng

**Affiliations:** ^1^ Department of Operating Room Nursing, Huai’an Maternal and Child Health Care Hospital Affiliated to Yangzhou University, Huai’an, China; ^2^ Department of Pediatric Surgery, Huai’an Maternal and Child Health Care Hospital Affiliated to Yangzhou University, Huai’an, China

**Keywords:** catheter fracture, pediatric emergency, retained fragment, venous access team, standardized protocols

## Abstract

**Background:**

Indwelling venous catheters, including peripheral intravenous catheters (PIVCs), are vital in pediatric care for delivering medications and fluids. However, catheter fractures, though rare (incidence 0%–2.1%), pose serious risks such as pulmonary embolism or cardiac arrhythmias if fragments migrate. While central venous catheter fractures are well-documented, PIVC fractures are underreported despite their widespread use.

**Case presentation:**

This report details two pediatric cases of PIVC fractures. In the first, a 1-day-old female neonate experienced a fractured left axillary catheter. Nursing staff promptly applied compression and immobilization, enabling successful surgical retrieval of a 3.5 cm fragment within 6 h, with no complications. In the second, a 1-year-old male infant had a right temporal vein catheter fracture, unrecognized for 6 days due to initial oversight, resulting in localized inflammation. CT angiography and ultrasound confirmed fragment locations, guiding surgical removal of a 1.5 cm fragment.

**Discussion:**

These cases highlight the rarity of PIVC fractures and the pivotal role of nursing vigilance in early detection—marked by signs like resistance during flushing or swelling. Timely interventions, such as compression and immobilization, prevent fragment migration and improve outcomes, as seen in the neonate, contrasting with the delayed case. Risk factors include excessive manipulation, improper site preparation (e.g., unshaved hair), and patient agitation. Preventive measures—thorough hair removal, secure fixation, daily inspections, and agitation management—are critical. Nursing education, standardized protocols, and vascular access teams enhance safety.

**Conclusion:**

PIVC fractures in pediatrics, though uncommon, demand nursing alertness and swift action. This series underscores the need for preventive strategies and training to optimize patient safety and outcomes.

## 1 Introduction

Indwelling venous catheters are indispensable in pediatric medicine, providing reliable vascular access for medications, fluids, and nutritional support while reducing repeated venipunctures (1). These devices include central venous catheters (CVCs), peripherally inserted central catheters (PICCs), and peripheral intravenous catheters (PIVCs), the latter encompassing scalp vein sets and butterfly needles, each serving specific clinical needs based on treatment requirements and patient characteristics (1).

Despite their benefits, catheter fracture is a rare but potentially life-threatening complication. Published studies report an incidence of 0%–2.1% for indwelling venous catheters, including central venous devices and peripheral intravenous catheters (PIVCs) in pediatric patients (2, 4). Although central line fractures are well-documented, PIVC fractures remain underreported despite their widespread use (2). Retained catheter fragments pose significant risks, including vessel perforation, thrombosis, infection, pulmonary embolism, and cardiac arrhythmias due to fragment migration (2).

This case report presents two distinct instances of retained catheter fragments in pediatric patients: a 1-day-old female neonate with a fractured left axillary indwelling catheter and a 1-year-old male infant with a retained fragment from a right temporal superficial vein catheter. These cases are particularly noteworthy as they involve peripheral venous access devices rather than central lines, which are more commonly reported in the literature (2). By contrasting outcomes between promptly recognized vs. delayed identification of catheter fractures, this report highlights the critical importance of nursing vigilance, prompt recognition, and effective management across all vascular access device types (3). Furthermore, it aims to enhance awareness of PIVC

fracture risk factors, illustrate essential nursing interventions for prevention and management, and advocate for comprehensive vascular access protocols and specialized training programs to mitigate such risks in pediatric practice.

## 2 Case presentation

### 2.1 Case 1: 1-day-old female neonate

**Figure 1:**
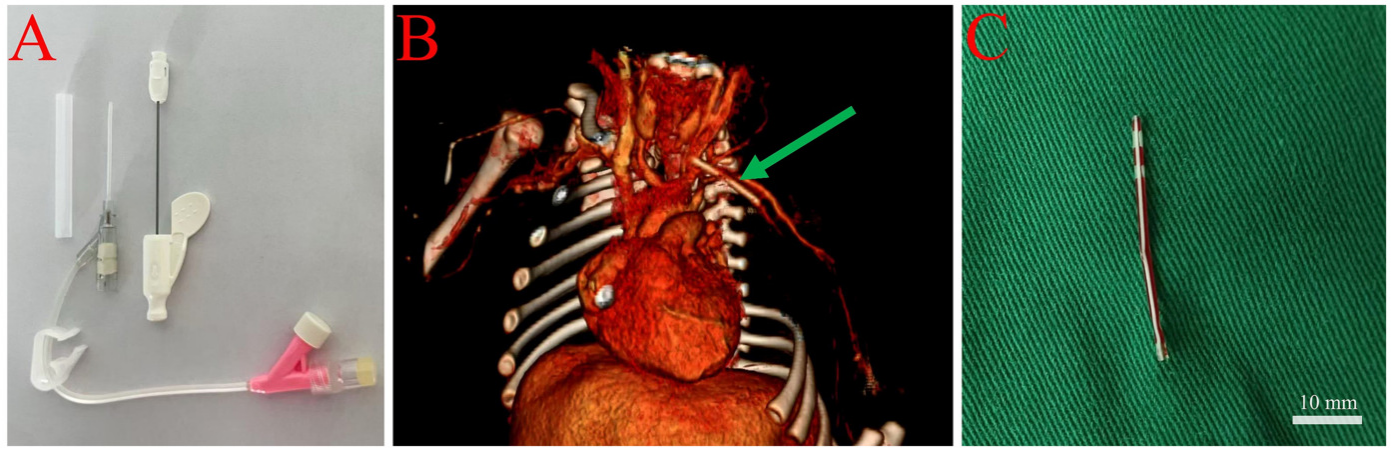
Management of catheter fracture in a 1-day-old female neonate. **(A)** Image of a newly secured 24G peripheral venous catheter in the distal limb, prepared for surgical intervention. **(B)** Three-dimensional computed tomography (CT) angiography illustrating the location of the fractured catheter (indicated by the green arrow) within the left subclavian and axillary veins. **(C)** Intraoperative image of the removed intact catheter fragment measuring 3.5 cm, with a scale bar, following surgical exposure and vascular clamping.

A 1-day-old female neonate was admitted to the neonatal intensive care unit (NICU) due to macrosomia associated with maternal diabetes. During routine venous cannulation via the left axillary vein, the indwelling catheter fractured, leaving a 3.5 cm segment retained *in situ* for 6 h (Figure 1A). Upon suspicion of catheter fracture, nursing staff immediately applied proximal compression to prevent fragment migration and positioned the infant in lateral decubitus on the affected side to minimize movement. The incident was documented, and the medical team was promptly notified. The infant was subsequently transferred to the emergency department.

On admission, physical examination revealed a puncture mark and mild local swelling in the left axillary region. Computed tomography (CT) angiography confirmed the presence of a foreign body in the left subclavian and axillary veins (Figure 1B). The nursing team prepared the infant for surgery by securing a new peripheral venous access using a 22–24G catheter in the right forearm (contralateral limb), flushing with minimal saline to maintain patency, and minimizing the infant's movement during preparation and transport.

Under general anesthesia, a 5 cm oblique incision was made to expose the axillary and subclavian veins. A palpable bulge was identified in the mid-axillary vein, and following vascular clamping, a 3.5 cm catheter fragment was successfully removed intact (Figure 1C). The vessel was repaired, and the incision was closed in layers. Postoperatively, the nursing team administered prophylactic antibiotics, monitored for signs of infection, and ensured routine postoperative care. The infant recovered well and was discharged 10 days after surgery. Follow-up assessments at 7 days, 1 month, 3 months, and 1 year post-discharge showed no complications.

### 2.2 Case 2: 1-year-3-month-old male infant

**Figure 2:**
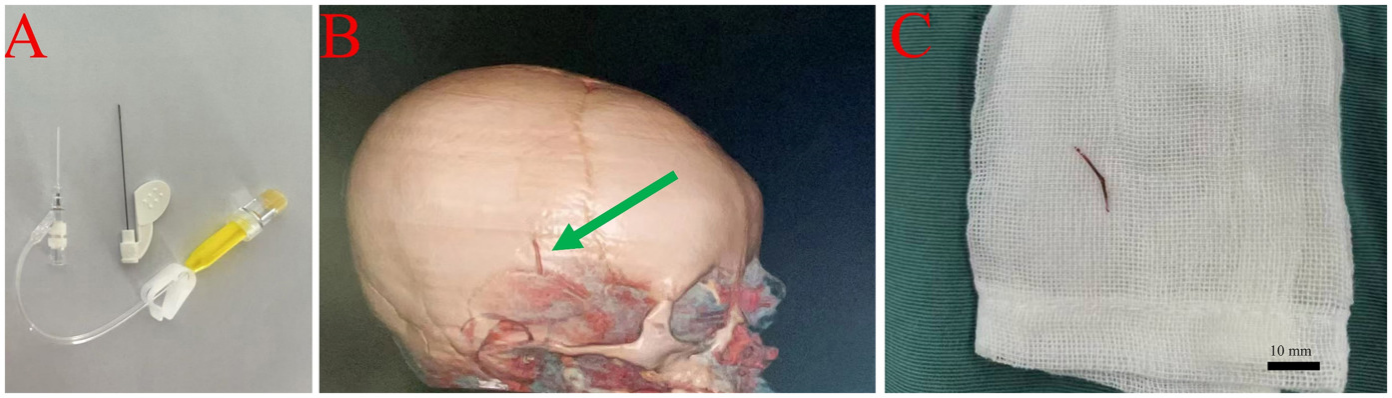
Removal of subcutaneous catheter fragment in a 1-year-3-month-old male infant. **(A)** Photograph of a newly inserted peripheral catheter at the affected site, prepared for surgical intervention. **(B)** Three-dimensional computed tomography (CT) angiography of the head, showing a 1.5 cm fractured catheter fragment within the right superficial temporal vein, as indicated by the green arrow. **(C)** Intraoperative photograph of the retrieved catheter fragment, measuring approximately 1.5 cm, with a scale bar, following surgical excision under general anesthesia.

A 1-year-3-month-old male infant was admitted with a 6-day history of a subcutaneous foreign body in the right temporal region. The incident occurred at a local pediatric hospital during intravenous therapy, when a 24G scalp vein set (butterfly needle, 19 mm length) in the right superficial temporal vein fractured during removal (Figure 2A).

The breakage was caused by the use of scissors to cut adhesive dressing that had adhered to unshaved hair. Initial ultrasound examination revealed no abnormalities, and no intervention was undertaken. Six days later, the family noticed redness, swelling, and a palpable cord-like structure approximately 1.5 cm in length in the right temporal area. CT angiography confirmed the presence of a foreign body in the right superficial temporal vein (Figure 2B).

Upon admission, nursing staff recognized the urgency of the case and prepared the patient for surgical intervention. The affected area was immobilized, and signs of further complications were closely monitored. Under general anesthesia, an incision was made along the margin of the swelling, and a 1.5 cm foreign body was successfully removed in its entirety (Figure 2C). Postoperatively, the nursing team administered prophylactic antibiotics and provided routine wound care. The infant recovered well and was discharged 2 days after surgery. Follow-up evaluations at 7 days, 1 month, and 3 months post-discharge revealed no complications.

## 3 Discussion

The occurrence of peripheral intravenous catheter (PIVC) fractures in pediatric patients is a rare yet potentially serious complication in clinical practice, with an estimated incidence ranging from 0 to 2.1% (2, 4). Unlike central venous catheter (CVC) complications, PIVC fractures are less frequently reported but can result in significant morbidity or even life-threatening events such as pulmonary embolism or cardiac arrhythmias if not promptly recognized (5, 6).

The iatrogenic fracture in Case 2, caused by cutting adhesive dressing with scissors in the presence of unshaved hair, underscores the importance of safe catheter removal techniques. Nurses should use adhesive removers or gentle manual separation to detach dressings, avoiding sharp instruments near insertion sites, particularly in agitated pediatric patients. Thorough hair clipping before insertion and standardized removal protocols can further mitigate such risks.

Nursing staff act as the first line of defense in detecting PIVC-related complications, given their constant proximity to patients and familiarity with catheter function. Early signs such as resistance during flushing, visible catheter damage, or localized swelling are critical indicators of catheter malfunction (7, 8). In the presented neonate, prompt identification of a catheter fracture by nurses, followed by immediate measures including proximal compression and limb immobilization, enabled the uneventful surgical retrieval of a 3.5 cm fragment, with no subsequent complications.In contrast, delayed recognition in another case led to a 6-day fragment retention and localized inflammation, illustrating the profound impact of timely nursing intervention on clinical outcomes.

Rapid and effective intervention is essential. Immediate actions, such as compression proximal to the fracture site and immobilization, are pivotal in preventing migration of the catheter fragment and reducing the risk of embolization or further complications (9, 10). Surgical retrieval of retained fragments, when indicated and executed promptly, is associated with better prognoses. In contrast, delays can result in adverse sequelae such as local inflammation, infection, or embolic events (11).

Diagnostic imaging plays a crucial role in confirming the presence and localization of retained PIVC fragments. Bedside ultrasonography offers a non-invasive and readily available modality for initial assessment, while computed tomography (CT) angiography provides superior spatial resolution, particularly when fragments are deeply embedded or migration is suspected. Both modalities were indispensable in our cases and in previously reported series, facilitating appropriate management decisions.

Several factors contribute to PIVC fractures in pediatric populations. These include excessive catheter manipulation, improper use of razors during site preparation, and agitation or uncontrolled movement of neonates and infants (12, 13). Inadequate training or inattention to secure fixation techniques further increases the risk of catheter damage. In our report and the reviewed literature, scalp vein catheters in neonates and infants were most commonly implicated, underscoring the vulnerability of this population.

Analysis of the case reports summarized in Table 1 reveals that PIVC fractures predominantly occur in pediatric patients, particularly those requiring scalp vein catheters. Of the 14 cases reviewed, 12 involved scalp venous indwelling needles in patients with an average age of 19.17 ± 8.96 months, highlighting the vulnerability of this young population. The primary causes of catheter fractures included improper use of razors during site preparation (8 cases), catheter folding (2 cases), and patient agitation (2 cases), underscoring the critical need for meticulous insertion techniques and secure fixation methods. Diagnostic imaging, such as bedside ultrasound and computed tomography (CT), played a pivotal role in confirming the presence and location of retained fragments, aligning with the importance of early and accurate detection discussed earlier. Treatment approaches varied from simple removal with hemostatic forceps to surgical interventions, with most cases achieving successful outcomes when addressed promptly. These findings reinforce the significance of nursing vigilance and immediate intervention, as delays in recognition and management can lead to complications such as localized inflammation or even embolic events, as seen in one case where a fragment was retained for six days.

**Table 1:** Summary of peripheral venous catheter fracture case reports retrieved from pubMed database over the past 20 years.

Indwelling Needle Type	Number of Cases	Age	Rupture Location	Ruptured Catheter Length (mm)	Rupture Cause	Diagnostic Method	Preventive Measures	Treatment
Scalp Venous Indwelling Needle (18)	12	19.17 ± 8.96 mo	Right forehead, Median frontal vein, Right superficial temporal vein	12–17	Improper use of razor (8 cases), Catheter folding (2 cases), Agitation during extubation (2 cases)	Bedside ultrasound, Ordinary CT, 3D-CT	Full shaving of hair, avoid excessive tape fixation, daily evaluation of puncture site	Squeeze out with hemostatic forceps (5 cases), First surgery (7 cases with 3D-CT or CT guidance), Second surgery (1 case with failed initial attempt)
Peripheral Venous Catheter (19)	1	1 day (preterm)	Right ventricle	15	Accidental fracture during cannulation	Echocardiography, Ultrasound, Chest x-ray	Avoid repeated needle reinsertion, check catheter integrity during removal	Surgical removal via median sternotomy under cardiopulmonary bypass (CPB)
Scalp Vein Indwelling Needle (Soft, flexible cannula) (20)	1	1yr	Scalp vein (near the skin)	Not specified	Movement of the child, improper fixation, excessive sweating	Multislice Spiral CT (MSCT), 3D Reconstruction	Skilled operation, proper fixation of needle, parents advised not to touch needle seat	Removal of the broken catheter; re-insertion into dorsal foot vein

Prevention must be prioritized through multiple strategies. Incorporating thorough hair removal (preferably by clipping, not shaving), secure but non-traumatic catheter fixation, and daily inspection of insertion sites can significantly reduce the incidence of catheter fractures (14). In addition, effective pain and agitation management, using comfort measures or pharmacological support, helps minimize inadvertent catheter dislodgement. Ongoing education and training for nursing staff on optimal catheter insertion, maintenance, and the early identification of complications are vital (15). The implementation of standardized institutional protocols for vascular access, as well as the introduction of innovative technologies such as advanced catheter materials and vein visualization devices, further enhances patient safety (16).

Furthermore, the preventive measures outlined in the case reports, including thorough hair removal and daily inspection of insertion sites, echo the recommendations for reducing catheter-related complications through standardized protocols and ongoing staff education.

The formation of specialized pediatric vascular access teams (VATs) has been linked to lower complication rates and improved outcomes, as these teams ensure standardized training and provide technical expertise to frontline staff. Institutional investment in such teams should be considered a best practice in modern pediatric care (17).

This report is limited by its dual case presentation and retrospective design, which inherently restricts generalizability. However, the purpose of this study is not to provide broadly applicable conclusions but to highlight the underrecognized risk of PIVC fractures in pediatric patients and promote awareness of preventive nursing strategies to enhance patient safety in such rare but critical situations. The insights provided reinforce the critical importance of nursing vigilance, prompt intervention, and structured preventive measures. Future research should focus on large-scale, prospective studies to better elucidate risk factors and evaluate the long-term efficacy of preventive strategies. Additionally, systematic follow-up of patients with retained fragments is warranted to assess the potential for delayed complications.

## 4 Conclusion

This case series reaffirms the indispensable role of nursing in the early detection, management, and prevention of PIVC fractures in pediatric patients. Through immediate action, adherence to evidence-based protocols, and integration of preventive measures into daily nursing practice, nurses can significantly improve patient safety and outcomes. The findings underscore the necessity of continuous education, institutional standardization, and the implementation of specialized vascular access teams to meet the complex demands of pediatric vascular care.

## Data Availability

The raw data supporting the conclusions of this article will be made available by the authors, without undue reservation.
